# Case Report: Munc13–4 deficiency presenting with autoimmune neuropathy years before FLH: implications for early genetic screening and HSCT timing

**DOI:** 10.3389/fimmu.2026.1890404

**Published:** 2026-07-20

**Authors:** Xiaojing Wei, Jianian Hu, Liye Bao, Hui Sun, Na Bai, Xuefan Yu, Jie Lin

**Affiliations:** 1 Department of Neurology and Neuroscience Center, the First Affiliated Hospital of Jilin University, Changchun, Jilin, China; 2 Center for Rare Diseases, The First Hospital of Jilin University, Jilin University, Changchun, China; 3 Department of Neuology, Huashan Hospital, Fudan University, Shanghai, China; 4 Huashan Rare Disease Center, Huashan Hospital, Fudan University, Shanghai, China; 5 National Center for Neurological Disorders, Shanghai, China; 6 Department of Emergency, Changchun Hospital of Traditional Chinese Medicine, Changchun, China; 7 Department of Electrophysiology, The First Affiliated Hospital of University of Science and Technology of China, Hefei, China

**Keywords:** autoimmune neuropathy, familial hemophagocytic lymphohistiocytosis, immune deficiency, perforinopathy, UNC13D

## Abstract

**Background:**

The *UNC13D* gene encodes Munc13-4, a critical regulator of cytotoxic granule exocytosis in lymphocytes and the protein responsible for familial hemophagocytic lymphohistiocytosis (FHL). As an essential mediator of perforin release from cytotoxic T and NK cells, Munc13–4 ensures immune surveillance by facilitating granule-plasma membrane fusion. Defects in this pathway, termed “perforinopathy“ manifest as impaired cytotoxicity and consequent immune dysregulation. While central nervous system involvement and systemic inflammation are established features of Munc13–4 deficiency, its association with autoimmune neuropathy remains largely unrecognized.

**Case report:**

We report two patients with a unique disease trajectory, presenting initially with autoimmune neuropathy before developing progressive FHL several years later. Both patients met the HLH-2004 diagnostic criteria, including fever, pancytopenia, hyperferritinemia, hypertriglyceridemia, and splenomegaly. Genetic analysis revealed heterozygous *UNC13D* mutations: in Case 1, a nonsense (c.3193C>T; p.Arg1065*) and a missense (c.1135G>A; p.Glu379Lys) mutation; in Case 2, compound heterozygous mutations comprising a maternal splicing variant (c.2447 + 1G>A) and a paternal missense mutation (c.1241G>T; p.Arg414Leu). Case 1 underwent hematopoietic stem cell transplantation (HSCT), with significant recovery of weakness and numbness, and no recurrence of fever or lymphadenopathy at one year. Case 2 succumbed to disease progression after no timely, suitable allogeneic hematopoietic stem cell donor could be found.

**Discussion:**

Our findings identify autoimmune neuropathy as a potential harbinger of FHL in perforinopathy, suggesting these neurological manifestations may be the initial phenotype of an underlying cytotoxic dysfunction. This temporal association mandates heightened clinical vigilance for immunodeficiency in patients with refractory neuropathy, particularly when accompanied by axonal degeneration or systemic inflammation. Recognizing this novel disease continuum has immediate diagnostic and therapeutic implications, as early genetic identification creates a critical window for potentially curative intervention with HSCT before irreversible multi-organ damage occurs. These cases expand the clinical spectrum of perforinopathy and challenge current diagnostic paradigms, emphasizing the need for an integrated neurological and immunological evaluation for unexplained autoimmune neuropathy.

## 1 Introduction

The *UNC13D* gene encodes the Munc13–4 protein, which is associated with familial hemophagocytic lymphohistiocytosis type 3 (FHL-3). While *PRF1* mutations (FHL-2) represent the most common genetic subtype, accounting for approximately 50% of cases, defects in *UNC13D*, *STX11*, and *STXBP2* (FHL-3, -4, and -5, respectively) all disrupt the intracellular trafficking apparatus required for targeted perforin delivery to the immune synapse (1–4). This shared pathophysiology underlies the unifying concept of “perforinopathy”, a spectrum of disorders characterized by either quantitative perforin deficiency or functional impairment in its delivery system (5).

As a key mediator of vesicle priming and synaptic membrane fusion, Munc13–4 ensures the precise release of lytic granules, a process indispensable for immune cell-mediated pathogen clearance, tumor surveillance, and homeostasis (2, 6, 7). Beyond this canonical role in cytotoxicity, emerging evidence implicates Munc13–4 in broader immune regulation, including the modulation of interferon signaling through endoplasmic reticulum-mediated mechanisms (6, 8). The clinical consequences of *UNC13D* deficiency are profound; impaired delivery of perforin and granzymes results in defective cellular cytotoxicity that is phenotypically indistinguishable from that caused by perforin mutations themselves (9). A previous study suggested that FHL-3 with low *UNC13D* levels triggers hyperinflammatory responses (2). The neurological implications are particularly noteworthy, as patients with variants frequently develop severe neuroinflammatory manifestations (10, 11). Recent evidence further suggests that hypomorphic mutations may predispose individuals to chronic inflammatory demyelinating polyradiculoneuropathy (CIDP) as an initial presentation (12). This observation raises a crucial question about the natural history of *UNC13D*-mediated disease: could autoimmune neuropathy represent a *forme fruste* of developing FHL-3? The current literature leaves this question unanswered, as no systematic studies have examined whether patients presenting with autoimmune neuropathy and biallelic *UNC13D* variants inevitably progress to full-blown FHL. This knowledge gap has significant clinical implications. Understanding the potential trajectory from isolated neuropathy to systemic HLH could revolutionize early detection and intervention for this life-threatening disorder. Our study addresses this need by characterizing the clinical evolution of *UNC13D*-related perforinopathy, with a specific focus on the temporal relationship between autoimmune neuropathy and subsequent FHL development.

## 2 Case description

### 2.1 Clinical case 1

**Figure 1:**
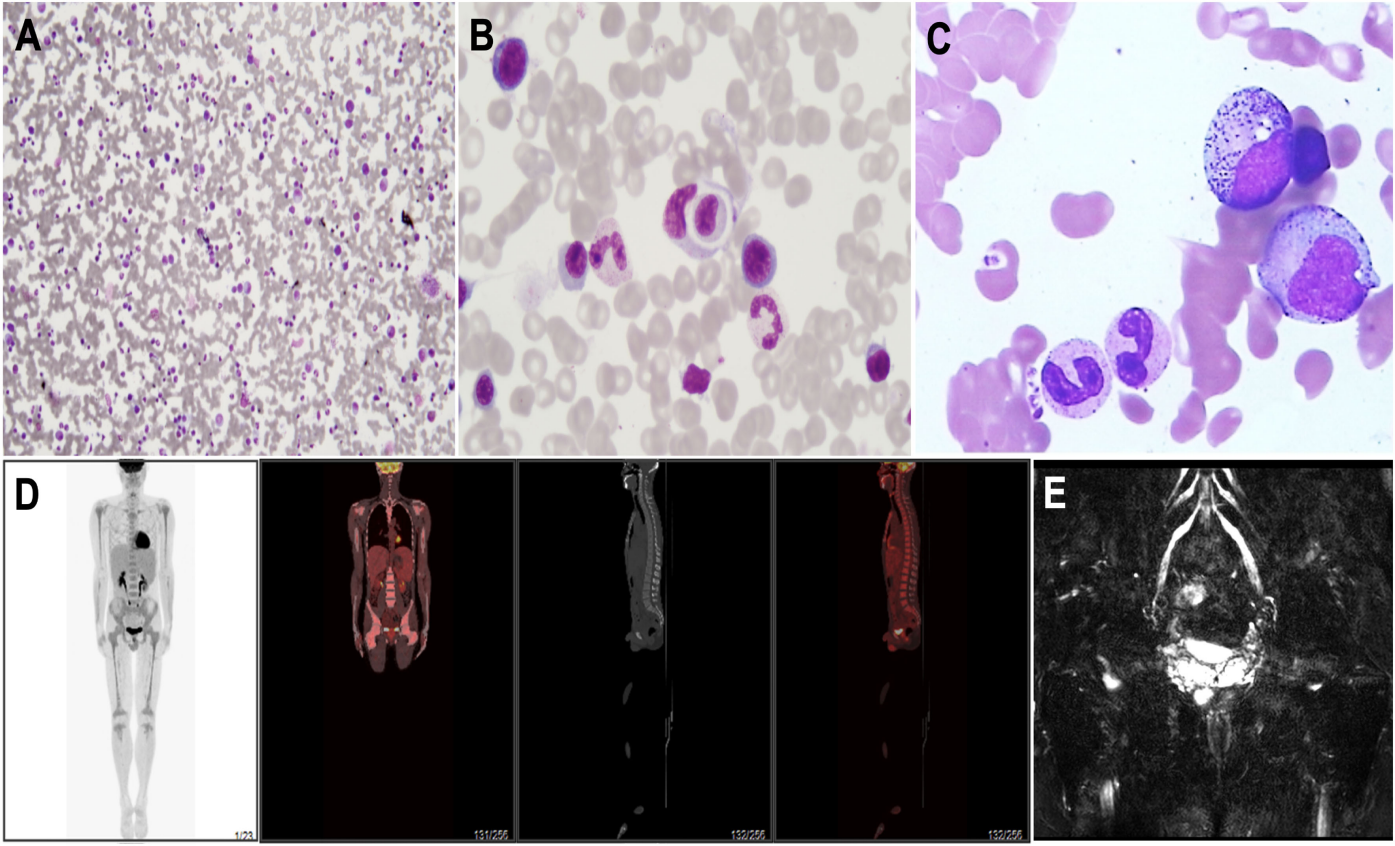
Bone marrow biopsy, PET-CT scan and neuroradiology study of the patients. **(A, B)** Hyperplastic bone marrow was observed in patient 1, with marked erythroid and megakaryocytic proliferation. The granulocytic lineage is reduced in proportion with partial left shift and some degenerative changes. Occasional atypical lymphocytes and hemophagocytic cells are observed on the smear. **(C)** In the bone marrow smear of case 2, the lymphocytes exhibited marked morphological irregularity, with some cells showing tail-like projections or pseudopodia. In addition, a small number of phagocytic cells. **(D)** Fluorodeoxyglucose-positron emission tomography/computed tomography (^18^F-FDG PET-CT) of patient 1 showed hypermetabolic lymphadenopathy and splenic uptake. **(E)** MRN of the lumbosacral plexus in case 2. MIP of a T2-weighted SPACE 3D sequence demonstrates diffuse enlargement (hypertrophy) and T2w-hyperintense signal of the lumbosacral plexus nerves, indicative of demyelination and inflammation. MIP, Maximum intensity projection; MRN, MR-neurography.

A 33-year-old male presented with progressive distal extremity numbness, heel burning pain, and impaired tactile sensation, causing his footwear to slip off unintentionally. Over five months, his symptoms worsened, with weakness progressing to limb paralysis that left him unable to walk unassisted. He reported no cranial nerve involvement. The patient subsequently developed recurrent fever, hepatosplenomegaly, and pancytopenia unresponsive to antibiotics. Upon admission, laboratory workup revealed negative serum immunofixation electrophoresis and normal VEGF levels. Cerebrospinal fluid (CSF) analysis revealed elevated protein (1, 135 mg/L) without pleocytosis, indicating neuroinflammation. Serum testing was positive for anti-NF186 antibodies. Electromyography (EMG) showed severely reduced motor and sensory nerve conduction velocities (MNCV/SNCV: 23–29 m/s). An initial bone marrow biopsy showed scattered hemolytic cells (**Figures 1A, B**), but the workup did not meet the criteria for hemophagocytic lymphohistiocytosis (HLH) at that time. After excluding POEMS syndrome and hematologic malignancy, a diagnosis of chronic inflammatory demyelinating polyneuropathy (CIDP) was made. He received intravenous methylprednisolone pulse therapy (500 mg daily for 5 days, then 240 mg daily for 3 days), followed by oral maintenance steroids, with partial improvement in strength.

Despite treatment, his neuropathy progressed to asymmetric limb weakness (MRC 2-4/5), areflexia, and profound sensory loss. After discontinuing oral steroids, he experienced worsening limb numbness and weakness accompanied by fever (39.8 °C), leading to readmission. He received methylprednisolone (80 mg daily for 3 days) followed by oral tapering, with improvement leading to discharge. His condition subsequently deteriorated with recurrent fever (39.8 °C), hepatosplenomegaly, and pancytopenia. Laboratory workup revealed hyperferritinemia (1, 011 ng/mL), hypofibrinogenemia (1.17 g/L), elevated triglycerides (3.7 mmol/L), and diminished natural killer (NK)/T cells, meeting the diagnostic criteria for HLH. A repeat bone marrow biopsy confirmed hemophagocytic cells. ¹^8^F-FDG PET-CT showed hypermetabolic lymphadenopathy and splenic uptake (**Figure 1D**), but a lymph node biopsy demonstrated only reactive hyperplasia without malignancy. Tests for Epstein-Barr virus (EBV) and tuberculosis were negative, and an autoimmunity panel (ANA, ENA) was negative. Given the lack of a clear etiology, genetic testing was initiated.

### 2.2 Clinical case 2

A 32-year-old male had a six-year history of progressive numbness in his hands and feet, accompanied by weakness, imbalance, and motor incoordination. Neurological examination revealed bilateral pes cavus, severe peroneal atrophy, generalized areflexia (except for weak biceps/triceps reflexes), and profound sensory loss in a stocking-glove distribution. Muscle strength was graded 2/5 in the upper limbs and 3/5 in the lower limbs, with foot drop due to ankle dorsiflexor weakness. EMG demonstrated severely slowed MNCV (18–27 m/s) and absent responses in multiple nerves. CSF analysis showed an increased protein level (1, 467 mg/L) with a normal white blood cell count. A T2-weighted SPACE 3D sequence demonstrated diffuse enlargement and T2w-hyperintense signal of the lumbosacral plexus nerves, indicative of demyelination and inflammation (**Figure 1E**). Despite steroid therapy, his symptoms persisted, indicating treatment-refractory disease.

Six years after the initial symptoms, he developed recurrent high fever (40 °C), hepatosplenomegaly, and abdominal lymphadenopathy. Laboratory tests revealed pancytopenia, hyperferritinemia (558.6 μg/L), hypertriglyceridemia (2.18 mmol/L), and hypofibrinogenemia (1.04 g/L). A bone marrow biopsy showed lymphocytosis (55%; **Figure 1C**) with abnormal CD3^+^/CD38^+^ T cells, and EBV viremia (>45, 000 copies/mL) was detected. His HScore of 251 indicated a 99% probability of HLH, fulfilling the HLH-2004 diagnostic criteria (13, 14). Genetic testing was performed to identify a potential underlying cause.

### 2.3 Genetic testing and diagnosis

Whole-exome sequencing (WES) was performed by Jiajian Genomics Lab (Beijing, China). Genomic DNA was extracted from peripheral blood samples using the DNA Midi Kit (Qiagen GmbH, Hilden, Germany). Exome libraries were prepared, captured, and sequenced on a NextSeq500 platform (Illumina, San Diego, CA, USA). Sequencing achieved 300× depth, covering 99.9% of the target regions. Raw reads were aligned to the human reference genome (hg19) using Burrows-Wheeler Aligner software. Single-nucleotide variants were identified and duplicate reads were marked using the Genome Analysis Toolkit (GATK). All steps followed standard WES protocols (15). Variants detected in probands were confirmed and segregated in available family members using Sanger sequencing.

**Figure 2:**
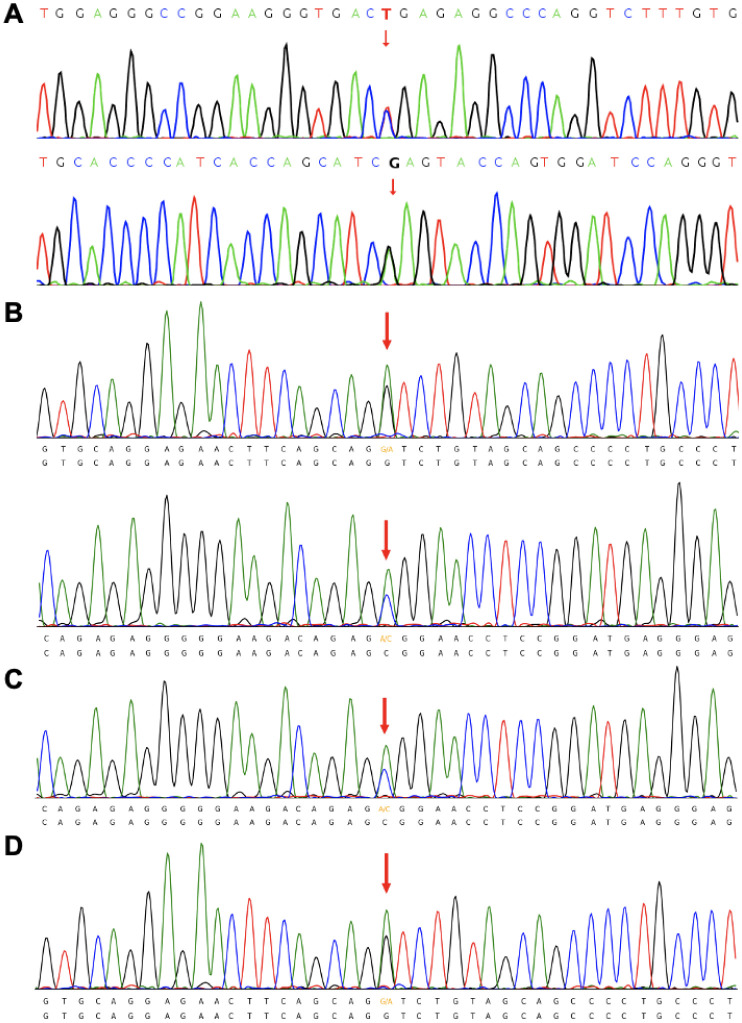
Mutation analysis. Sanger sequencing of DNA of patient 1 **(A)** showed heterozygous nonsense variation (c.3193C>T; p.Arg1065*) and heterozygous missense variation in the *UNC13D* (c.1135G>A; p.Glu379Lys). Sequencing of patient 2 **(B)**, his father **(C)** and mother **(D)** revealed a maternally inherited splice-site variant (c.2447 + 1G>A) and a paternally inherited missense mutation (c.1241G>T, p.Arg414Leu).

In Patient 1, WES identified compound heterozygous *UNC13D* mutations: a nonsense variant (c.3193C>T; p.Arg1065*) and a missense variant (c.1135G>A; p.Glu379Lys) (**Figure 2A**). Both mutation sites have been previously associated with FHL-3 (6, 9, 13–16). Segregation analysis confirmed that the two variants were inherited from each parent separately. The patient’s clinical history, manifestations, and laboratory findings confirmed a combined diagnosis of autoimmune neuropathy and FHL-3. The patient was admitted to the Department of Hematology and underwent hematopoietic stem cell transplantation (HSCT). At one year post−transplantation, weakness and numbness had markedly improved, with residual paresthesia confined to the feet. Clinical evaluation revealed a total INCAT score of 1 (upper limbs 0, lower limbs 1). No recurrent fever or lymphadenopathy was reported. In Case 2, genetic testing revealed compound heterozygous *UNC13D* mutations: a maternally inherited splice-site variant (c.2447 + 1G>A) and a paternally inherited missense mutation (c.1241G>T; p.Arg414Leu) (**Figures 2B–D**). Both have been previously linked to FHL-3 (16–18), explaining his refractory autoimmune neuropathy as a neurological manifestation of the disease. During initiation of the HLH-2004 therapy, the lack of a suitable allogeneic hematopoietic stem cell donor led to disease recurrence, severe multiple organ failure, and a fatal outcome.

### 2.4 Analysis of genetic variants

**Figure 3:**
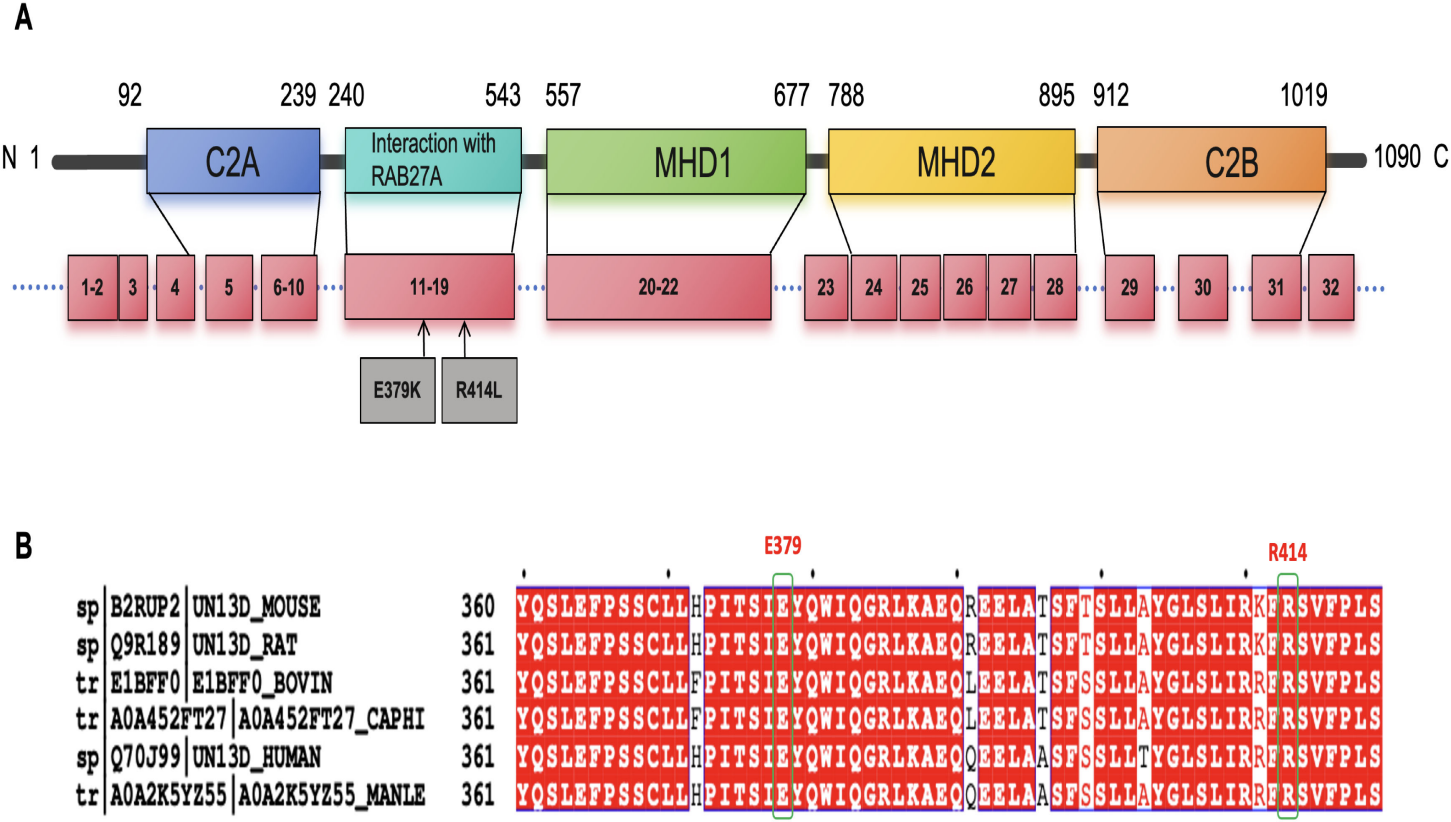
Localization of the mutations in *UNC13D* described in this study relative to exon structure and protein domains. **(A)** Sites of mutations described in this report relative to exon structure and protein domains: C2 (C2A and C2B) and Munc homology domains (MHD1 and MHD2). **(B)** Homology Model of Human *UNC13D* Protein and Respective Location of the Rare Variants Identified in This Study.

**Figure 4:**
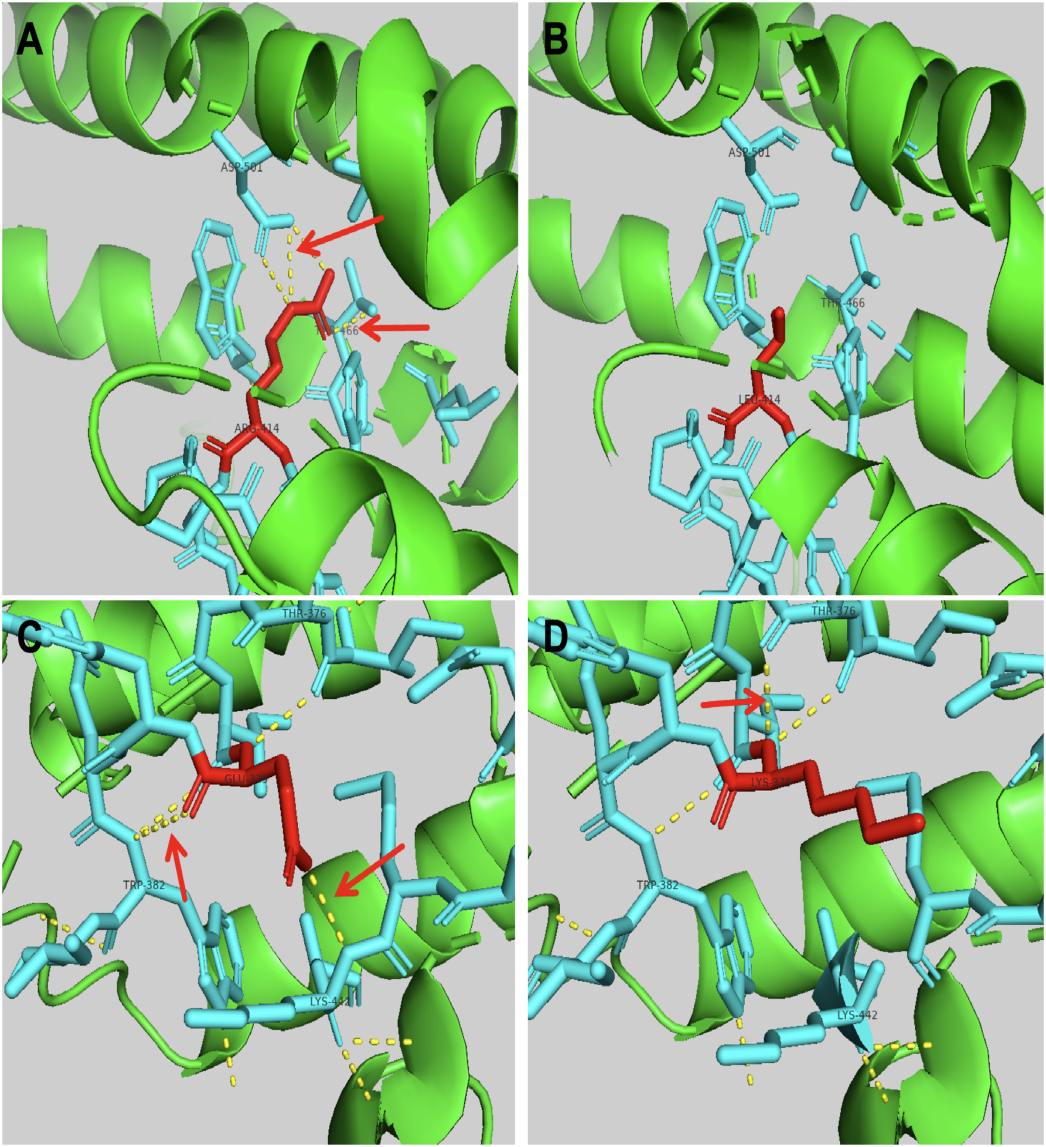
Location of amino acid mutations in patients. The bound GTP analog (GppNHp) is shown as a light blue stick modeland. Insets magnify the regionswhere mutations in patients, with R414L **(A, B)** and E379K **(C, D)** shown.

The identified variants and their locations are summarized in **Figure 3A**. The p. Arg414Leu mutation affects a highly conserved residue within the RAB27A-binding region, located between the N-terminal C2A and Munc13 homology domains (MHD1) (**Figure 3B**). This variant has been previously reported as pathogenic. Wild-type Arg414 forms critical hydrogen bonds with Asp501 and Thr466; mutation to a non-polar leucine disrupts these bonds, which is predicted to destabilize the local protein conformation and impair structural integrity (**Figures 4A, B**). Although this specific mutation has not been functionally assessed, Noori et al. evaluated the same-position variant p. Arg414Cys (R414C) and demonstrated that it retains approximately 20% of wild-type activity (19). Our structural predictions are consistent with previous reports showing loss of Munc13–4 protein expression resulting from the Arg414Leu mutation (17, 18), and the partial residual activity observed for R414C further suggests that mutations at this critical residue may lead to a hypomorphic (partial loss-of-function) rather than completely null phenotype. The Glu379Lys (E379K) variant also maps to a conserved RAB27A-binding region. Wild-type Glu379 forms hydrogen bonds with Trp382 and Lys442. Substitution with lysine abolishes these interactions but forms a new bond with Thr376, altering the conformational stability (**Figures 4C, D**). Furthermore, in silico analysis with PolyPhen-2 classifies it as “Probably Damaging”. Consistent with our predictions, Noori et al. experimentally demonstrated that this mutant retains residual activity despite reduced stability, suggesting a partial loss-of-function mechanism (19). Both E379K and R414L are recorded in the gnomAD database at very low allele frequencies (E379K: 4.96×10^-6^; R414L: 3.72×10^-6^), consistent with their classification as rare variants.

### 2.5 Identification expression levels of *UNC13D* in polyneuropathies-related differentially expressed genes

To investigate the relationship between *UNC13D* and peripheral neuropathy, we analyzed the expression profile of *UNC13D* in immune cells within polyneuropathy tissues. We leveraged previously published single−nucleus transcriptomic data (GSE285983) from peripheral nerves of 33 patients with polyneuropathies and 4 healthy controls (20), and visualized the results based on the primary analyses reported in the original publication. The result revealed a striking coherence between our findings and the previously reported expression patterns of *UNC13D* in immune cells linked to cytotoxic dysfunction-specifically, both data consistently demonstrated that aberrant *UNC13D* expression (e.g., upregulation in NK/T cells of polyneuropathies patients, as observed in our differential expression analysis) (**Supplementary Figure 1**).

This cross-validation not only strengthens the reliability of our observations but also provides further evidence for the role of *UNC13D*-mediated immune dysregulation in driving polyneuropathies-related damage.

## 3 Discussion

Familial hemophagocytic lymphohistiocytosis (FHL) is a congenital immunodeficiency caused by defects in cytotoxic granule exocytosis, a pathway termed “perforinopathy”. Mutations in genes such as *PRF1, UNC13D, STX11*, and *STXBP2* disrupt perforin delivery to immune synapses, leading to defective cytotoxicity, prolonged lymphocyte activation, and excessive cytokine production (5, 6). Central nervous system (CNS) involvement is common in HLH (21). Beken et al. reported that 44% of patients had CNS involvement at diagnosis, and an additional 30% developed CNS involvement during follow−up (22). In Chinese pediatric patients with HLH, the proportion of CNS involvement reached as high as 60.3% (23). Isolated central nervous system hemophagocytic lymphohistiocytosis (CNS-isolated HLH) is a rare but increasingly recognized phenotype in which neurological manifestations precede systemic involvement and occur without overt systemic inflammatory features; pathogenic variants in *PRF1* and *UNC13D* are the predominant genetic causes of this subtype (24–26). Accumulating evidence indicates that CNS-isolated HLH frequently mimics primary demyelinating disorders such as multiple sclerosis, leading to diagnostic delays and misdiagnosis (11). Pediatric case studies have further characterized the clinical trajectories of this entity and underscored the need for early genetic testing in patients with unexplained, treatment-refractory CNS inflammatory presentations (21, 26). Patients with CNS−isolated HLH typically show decreased or absent NK cell activity (25).

*UNC13D* loss-of-function variants, encompassing both coding missense mutations and intronic regulatory mutations, have emerged as important genetic susceptibility factors for a broad spectrum of immune-mediated diseases, such as myositis, autoimmune lymphoproliferative syndrome, systemic juvenile idiopathic arthritis, macrophage activation syndrome and autoimmune CNS disorders (6, 27–32). Mechanistically, these variants disrupt the granule exocytosis pathway, impairing the cytotoxic function of NK cells and CD8^-^ T lymphocytes, which leads to persistent immune dysregulation. This immune dysregulation not only triggers systemic inflammation but also renders patients susceptible to autoimmune complications such as neuropathy, an observation consistent with previous reports that have linked *PRF1* variants, which similarly disrupt perforin delivery to immune synapses as *UNC13D*, to CIDP (12). However, no prior studies have established an association between *UNC13D* mutations and peripheral neuropathy. Our research identifies a novel clinical trajectory: recessive *UNC13D* mutations may initially manifest as autoimmune neuropathy, preceding the onset of classical FHL symptoms by several years. This finding thereby expands the known spectrum of neurological complications associated with perforinopathy. Nevertheless, given the cross-sectional nature of our observation, this correlation between *UNC13D* variants and autoimmune neuropathy warrants further validation in larger prospective cohorts and functional *in vitro* studies.

The precise mechanisms linking perforinopathy to neuroinflammation remain to be fully elucidated. Notably, clonal expansion of cytotoxic T cells has been observed in the blood and peripheral nerves of patients with polyneuropathies (33, 34), while polyneuropathies have also been identified in those affected by “perforinopathy” (35, 36). As expected, single-nucleus transcriptomics of peripheral nerves revealed mild upregulation of *UNC13D* in distinct immune cell subsets of patients with polyneuropathies compared to healthy controls (20). We hypothesize that impaired NK/T-cell cytotoxicity creates a permissive environment for autoimmune dysregulation. This is supported by Case 2, in which concurrent autoimmune neuropathy and myelitis showed transient improvement during immunochemotherapy for HLH.

This temporal relationship strongly implicates cytotoxic lymphocyte dysfunction as the fundamental driver of neuroinflammation in these patients. While peripheral nerve involvement in HLH is rarely reported (12, 37–40), our findings position FHL at the severe end of a clinical spectrum where autoimmune neuropathy may serve as the initial presentation of an underlying perforin pathway defect. This temporal progression from isolated neuropathy to systemic FHL raises critical pathophysiological questions. We propose two potential mechanisms for this compartmentalized onset: Localized Immune Dysregulation: Neurological infections may trigger confined cytotoxic T/NK cell activation within the neural microenvironment; Subclinical Systemic Inflammation: Hypomorphic mutations (e.g., in *UNC13D* or *PRF1*) may preserve residual cytotoxic function, mask systemic manifestations while permitting low-grade immune activation. The pleiotropic roles of *UNC13D*, which regulates broader lysosomal transport beyond perforin release, may account for the frequent multisystem involvement in FHL-3 (6).

This biological complexity creates significant clinical challenges. Diagnosing FHL is often delayed, and when neuropathy precedes systemic symptoms, the diagnosis is frequently limited to the neurological condition. Immunotherapy for neuropathy may mask early signs of HLH, causing clinicians to miss the optimal window for intervention, as tragically demonstrated by our fatal case. Early recognition of this association is therefore paramount. The inexorable progression of FHL in genetically susceptible individuals suggests that autoimmune neuropathy can serve as a sentinel manifestation. Neurologists evaluating patients with refractory autoimmune neuropathy (especially the axonal subtype) who have one or more red flags-poor/transient response to standard immunotherapies, concurrent/recurrent systemic inflammatory signs, frequent relapses, or rapidly progressive axonal degeneration on electrodiagnostic testing-should adopt a sequential screening to rule out early FHL. First-tier HLH screening includes laboratory tests (serum ferritin for ≥500 ng/mL hyperferritinemia, triglycerides for ≥2.0 mmol/L hypertriglyceridemia, fibrinogen for ≤1.5 g/L hypofibrinogenemia, complete blood count for pancytopenia, NK cell cytotoxicity assay for functional defects), bone marrow biopsy to identify hemophagocytic cells, and HScore clinical scoring. Second-tier immunogenetic testing uses targeted gene panel sequencing or WES focusing on perforinopathy-related genes (*UNC13D, PRF1, STX11*, and *STXBP2*), with identification of biallelic pathogenic variants (compound heterozygosity/homozygosity) confirming the diagnosis.

In conclusion, these two cases underscore the critical but underrecognized link between *UNC13D* mutation and neurological manifestations-with autoimmune neuropathy as the initial clinical phenotype. Given its treatable nature, early recognition of this entity (prior to irreversible multi-organ damage) is critical to prioritize allogeneic hematopoietic stem cell transplantation (HSCT)-the sole curative modality for FHL to date-for patients with confirmed genetic defects.

## Data Availability

The datasets presented in this study can be found in online repositories. The names of the repository/repositories and accession number(s) can be found in the article/**Supplementary Materials**.
