# Supplementary material for: Case Report: Munc13–4 deficiency presenting with autoimmune neuropathy years before FLH: implications for early genetic screening and HSCT timing
Source: Front Immunol. 2026 Jul 20;17:1890404. doi: 10.3389/fimmu.2026.1890404 (PMC13429461; doi:10.3389/fimmu.2026.1890404)
Supplement: Supplementary file 1 [file DataSheet1.docx]

**
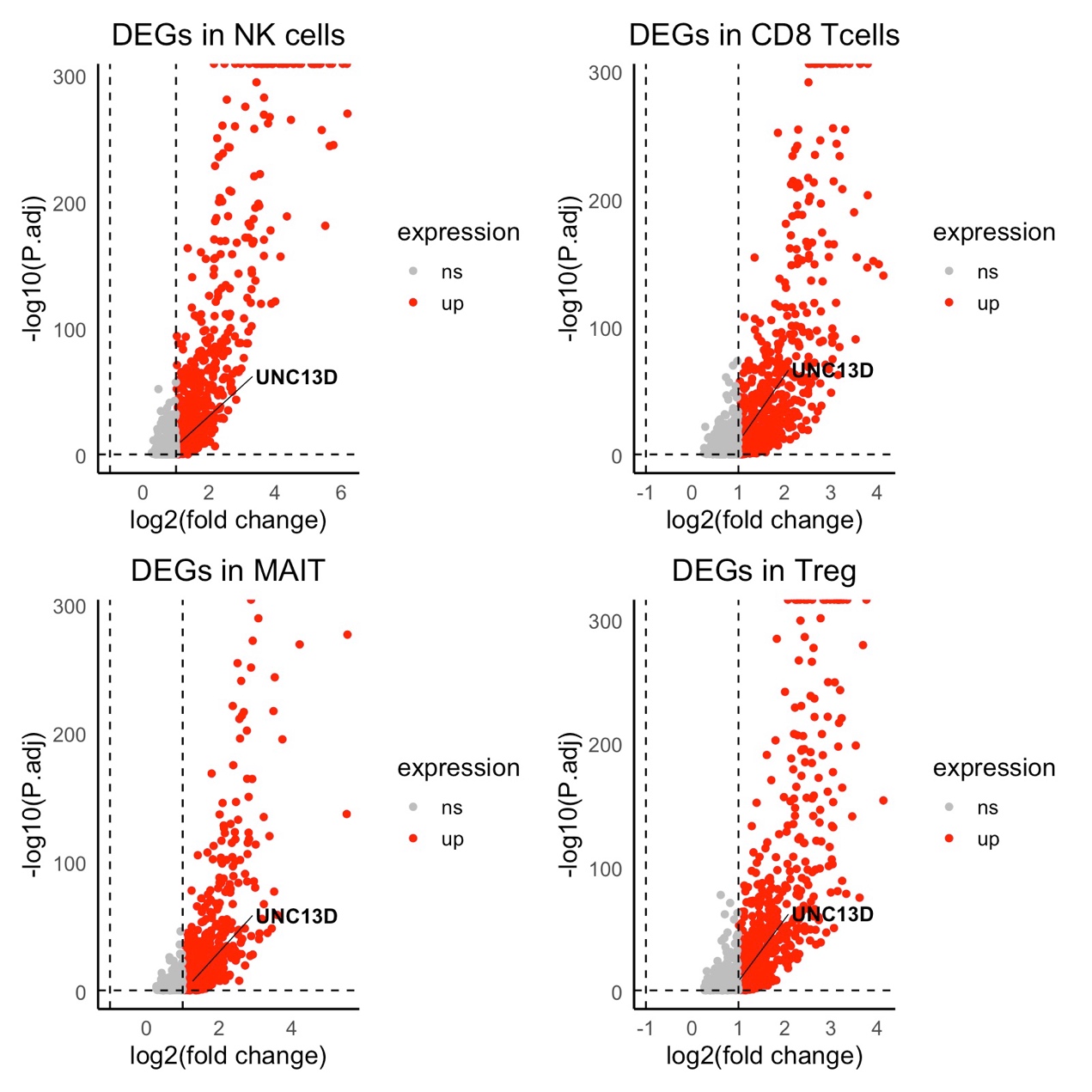
Supplementary Figure 1** Volcano plots showing that *UNC13D* in DEGs between polyneuropathies and healthy controls. The red spots indicate upregulated genes, whereas the gray spots indicate not significant genes.
